# Negative pressure pulmonary edema following laryngospasm after dental abscess: A case report

**DOI:** 10.1016/j.heliyon.2024.e28470

**Published:** 2024-03-24

**Authors:** Ali Hossein Samadi Takaldani, Kaveh Latifi, Asma Salmani, Mohammad Negaresh

**Affiliations:** a Department of Internal Medicine (Pulmonology Division), School of Medicine, Ardabil University of Medical Sciences, Ardabil, Iran; b Department of Anesthesiology and Pain Medicine, School of Medicine, Iran University of Medical Sciences, Tehran, Iran; c Department of Internal Medicine, School of Medicine, Ardabil University of Medical Sciences, Ardabil, Iran

**Keywords:** Pulmonary edema, Laryngospasm, Periapical abscess, Oral hygiene

## Abstract

Negative pressure pulmonary edema (NPPE), also known as post-obstructive pulmonary edema, is a rare and life-threatening condition. It occurs when a person breathes against an obstructed glottis, causing negative thoracic pressure in the lungs. This negative pressure can lead to fluid accumulation in the lungs, resulting in pulmonary edema. The obstructed glottis might be caused by laryngospasm, which occurs when the muscles around the larynx involuntarily spasm and can lead to complete upper airway occlusion. This report shares the case of a 33-year-old woman hospitalized for periapical dental abscess, facial swelling, and shortness of breath. The patient exhibited signs of poor oral hygiene. After the exacerbation of her symptoms, she showed signs of asphyxia and decreased oxygen saturation, which led to her intubation. Imaging revealed bilateral pleural effusion and patchy ground glass opacities favoring NPPE. After three days of treatment with diuretics and other conservative measures, her condition was alleviated, and she was extubated. Laryngospasm in the presence of a dental abscess is uncommon. Identification of imaging favoring NPPE in this setting is even more rare. In cases of laryngospasm, prompt intubation is crucial. Therapy with diuretics and other conservative measures can effectively treat NPPE following laryngospasm.

## Highlights

• Maintaining good instrument hygiene is crucial in the field of dentistry.
• In certain cases, dental abscesses may provoke an inflammatory process and cause laryngospasm.
• NPPE following a laryngospasm is an uncommon occurrence but should be considered in patients with findings of edema as it responds tremendously to treatment.

## 1 Introduction

A periapical abscess is a common type of dental abscess and significantly impacts the alveolar bones. Typically, these abscesses occur at the apex of the dental root, the tooth's periodontal membrane, or the adjacent alveolar bone. The spread of infection through the apical foramen triggers an inflammatory reaction that attracts a host of chemical mediators, initiating the periapical pathology. This cascade of events can ultimately lead to the formation of a periapical abscess, radicular cyst, or periapical granuloma [1,2]. Negative pressure pulmonary edema (NPPE), also known as post-obstructive pulmonary edema, is a rare and life-threatening condition that follows the vigorous relief of upper airway obstruction during extubation in a spontaneously breathing patient after general anesthesia or negative thoracic pressure induced by inspiratory effort against an obstructed glottis [3,4]. However, it can be caused by laryngospasm, epiglottitis, tumors, obesity, hiccups, or obstructive sleep. Tachypnea, coughing, pink frothy sputum, bilateral fluffy infiltrates on the chest radiograph, and the inability to maintain oxygen saturation above 95% are common presenting signs that may be mistaken for pulmonary aspiration or pulmonary embolism and can cause different diagnostic difficulties, especially for the anesthesiologist [5,6]. A limited number of cases have been documented wherein NPPE has been observed following maxillofacial surgeries during intubation or the surgical procedure [7,8]. The occurrence of laryngospasm in the presence of a dental abscess followed by NPPE imaging findings is uncommon. Treatment is generally supportive by maintaining a patent upper airway and oxygen supplementation, as most cases of NPPE resolve without medical intervention within 12–48 hours. Mechanical ventilation may sometimes be necessary for a short period [3]. We present a case of NPPE after laryngospasm due to periapical dental abscess.

## 2 Case presentation

**Fig. 1:**
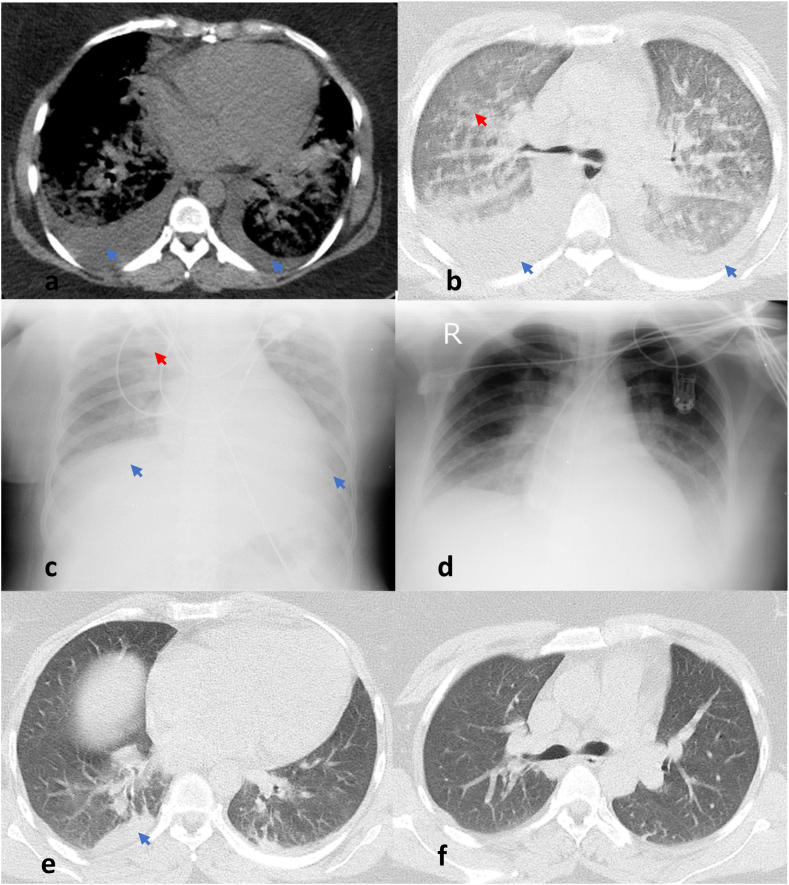
Patient's chest Imaging. a, b, chest CT scan at the first day of admission after laryngospasm displaying bilateral pleural effusion (Blue arrow) and bilateral ground glass opacities (Red arrow). c, d, chest radiography at the second and third day of admission. (For interpretation of the references to colour in this figure legend, the reader is referred to the Web version of this article.)

We present a 32-year-old woman referred to the hospital due to facial swelling and shortness of breath. Her lower left incisor, molar, and premolar teeth were extracted at a private dentist's office at 8 p.m. On the next day, at 10 a.m., she woke up with pain in the left jaw and swelling that gradually expanded to the left side of her cheek and neck until, at noon (2 p.m.), she reported experiencing shortness of breath after walking approximately 100 m, which caused her to stop and catch her breath before continuing, hoarseness, and fever, and she reached out to the hospital. She had a history of polycystic ovaries but mentioned no medical history in her family. Upon admission, she denied using any drugs, either daily or after the dental surgery. Her blood pressure was 160/80 mmHg, respiratory rate was 28 per minute, pulse rate was 125 beats per minute, and body temperature was 38 °C. She had an oxygen saturation (SpO2) of 58% in the room air, which increased to 85% after receiving 100% oxygen via a reservoir bag and being placed in a sitting position. In the physical examination, her appearance showed swelling and local heat in the left temporomandibular joint (TMJ) and masseter, and her face was asymmetrical. Upon examining the patient's oral cavity, she had poor oral hygiene, and other teeth on the right side required dental treatment. A tooth located in the upper right molar area had been extracted, and the space was empty. Her left mandibular region was tender, and two mobile, tender lymph nodes were palpable in the left submandibular and anterior cervical regions with an approximate size of 1.5 × 1.5 cm. In lung auscultation, crackles and decreased breathing sounds were detectable in the base of the lungs. Due to her condition, she was immediately transferred to the intensive care unit (ICU). The arterial blood gas analysis (ABG) at the ICU showed PH 7.25, PaO2 81%, PaCO2 43.2, and HCO3 18.1. She suddenly experienced tachycardia, increased respiratory rate, cyanosis, gasping respiration, use of accessory muscles, hypoxemia, and her SpO2 decreased to 75% with 100% o2 with the reservoir bag mask; her auscultation showed diffuse wheeze and crackle along with reduced breath sounds in lower lungs. This sudden worsening of her condition led to intubation. The patient was intubated after receiving 100 mcg of fentanyl and 2 mg of midazolam. During intubation, the operator claims proximity of the vocal cords and bucking during the endotracheal tube (ETT) passage, along with pink and frothy secretions coming out of the ETT. Her Spo2 after intubation, despite receiving Fio2 100%, increased to 85%. A lung CT scan showed bilateral pleural effusion and patchy ground glass opacities (Fig. 1a and b). These findings suggested a viral or bacterial infection, cardiogenic pulmonary edema, or non-cardiogenic pulmonary edema as differential diagnoses. The treatment began with 40 mg of furosemide amp as a bolus dose and then an infusion of 8 mg/hr., 1 g of vancomycin amp two times a day, and 600 mg of clindamycin three times a day. During her cardiac consultation and echocardiography, the cardiologist reported an ejection fraction of 55%, a pulmonary artery pressure (PAP) of 42 mm Hg, and up to moderate posterior mitral valve regurgitation. The cardiologist expressed that cardiac pathologies were less likely based on these findings. All workups for connective tissue diseases, vasculitis, COVID-19, and influenza were negative (Table 1).

**Table 1:** The laboratory results of the patient during her admission.

Lab tests		Normal range	Day 1	Day 2	Day 3	Day 4	Day 5	Day 6	Day 7
WBC count, count/mm		4000–10000	10.100		9900				5800
Hemoglobin, mg/dl		12–15	13.6		11.6				12.4
MCV, fl		80–96	87		85				85
ESR, mm/hr		>21		26					
CRP		Neg	Neg.						
Platelet, count/mm		150000–450000	303.000		216.000				
BUN, mg/dl		15–45	56	55	62	61		62	59
Cr, mg/dl		0.5–1.4	1.1	1.3	1.2	1.5		1.2	1.2
INR		1–1.4	1.1		1.3				
Troponin, ng/mL		0–0.04	0.039	0.03					
TSH, micIU/ml		0.35–4.94		1.2				2.3	
LDH, U/L		0–500		539					
AST, IU/l		<41		20					
ALT, IU/l		<41		24					
CPK, mcg/L		0–120		185					
ALP, IU/l				120					
ds-DNA		Neg.	Neg.						
Blood culture		Neg.	Neg.						
Urine culture		Neg.	Neg.						
C3, mg/dl		75 to 175	113.8						
C4, mg/dl		16 to 48	22						
CH50, U/mL		42 to 95	110						
ANA		<0.5	0.1						
P-ANCA		Neg.	Neg.						
C-ANCA		Neg.	Neg.						
D-dimer		Neg.	Neg.						
24 hr. urine	Cr. Pr. Vol.		13.2						
			592						
			1600						

**WBC**: White blood cells; Hb: Hemoglobin **MCV**: Mean corpuscular volume; **LDH**: Lactate dehydrogenase; **Cr**: Creatinine; **CRP**: C-reactive protein; **ESR**: Erythrocyte sedimentation rate; **INR**: International normalized ratio; **PTT**: Partial thromboplastin time; **BUN**: Blood urea nitrogen; **TSH**: Thyroid-stimulating hormone; **AST**: Aspartate aminotransferase; **ALT**: Alanine aminotransferase; **ALP**: Alkaline phosphatase; **CPK**: Creatinine phosphokinase**; ANA**: Anti-nuclear antibody; **ANCA**: Antineutrophil cytoplasmic antibodies; **ds-DNA**: Double stranded deoxyribonucleic acid.

Over the next few days, the patient's condition gradually improved. Her fever, as well as the swelling in her face and neck, began to subside. Her Spo2 levels reached 94% with 60% fio2. Chest radiography revealed decreased lung involvement on the second and third days (Fig. 1c and d). By the third day, she was weaned off the mechanical ventilator and transferred to the general ward on the fourth day of admission. On the sixth day, a spiral chest CT scan showed a minimal right pleural effusion (Fig. 1e and f). Since her dyspnea improved and her SpO2 without oxygen was 96%, she was discharged on the seventh day. Two weeks later, the patient revisited the pulmonologist and was found to be fully recovered. Please refer to Table 1 for the results of the laboratory test.

## 3 Discussion

Dental trauma, caries, and poor oral hygiene often cause dental abscesses. When the protective enamel of the teeth breaks down, bacteria from the mouth can enter the tooth cavity (known as the pulp cavity), causing an infection. As this infection grows within the pulp cavity, it can cause severe pain by compressing the inner dentine walls. The infection can then spread downwards through the root canal into the mandible or upwards into the maxilla, depending on the location of the affected tooth. Another cause is a partially erupted tooth, usually a wisdom tooth, where bacteria get trapped between the crown and soft tissues, causing inflammation. Other causes include genetic factors like amelogenesis imperfecta, mechanical factors, medical conditions like Sjogren's syndrome, Chemical irritants like smoke from methamphetamine, and immunosuppression due to chemotherapy or chronic diseases like HIV/AIDS [9,10].

**Fig. 2:**
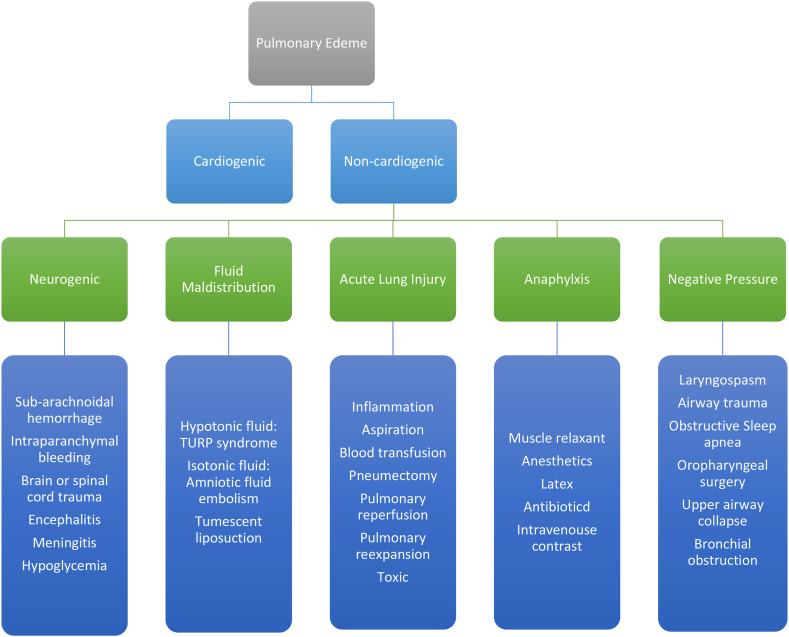
Differential diagnosis of acute pulmonary edema [12].

The accumulation of extravascular fluid in the lung parenchyma is known as pulmonary edema. This abnormal buildup can hinder the exchange of gases at the alveolar level and, in severe cases, lead to respiratory failure. This condition can be brought on by cardiogenic causes with the inability to remove enough blood from the pulmonary circulation, or it may stem from lung tissue damage known as non-cardiogenic etiologies (Fig. 2) [11,12].

In our case, the severity of the respiratory failure, the time course of clinical and radiologic findings, the normal cardiologic evaluation, and the fast resolution of the pleural effusion and pulmonary edema were not ultimately consistent with these etiologies and favored the NPPE diagnosis. The etiologies for NPPE are presented in Fig. 2. Based on the information presented, it is highly likely that NPPE resulted from a laryngospasm caused by an infection in the upper respiratory tract due to a periapical dental abscess.

NPPE was first demonstrated by Moore in 1927 in spontaneously breathing dogs subjected to resistive loading and is a rare noncardiogenic acute pulmonary edema that can be fatal if not diagnosed and treated promptly. For this reason, before diagnosing NPPE, other possible causes of acute pulmonary edema must be considered [13,14]. The pathophysiology of NPPE includes the creation of high negative intrapleural pressure through forceful inhalation against an obstructed upper airway in spontaneously breathing patients. This high negative intrapleural pressure augments blood flow to the right side of the heart, which in turn dilates and increases the hydrostatic pressure gradient across the pulmonary vascular bed, promoting the movement of fluid into the interstitial and alveolar spaces from the pulmonary capillaries. This negative pressure also increases the left ventricle afterload, thus decreasing the ejection fraction, which heightens left ventricular end-diastolic pressure, left atrial pressure, and pulmonary venous pressure, escalating the development of pulmonary edema via an increase of pulmonary hydrostatic pressure. Additionally, this negative pressure triggers activation of the sympathetic nervous system, hypertension, and central displacement of blood volume. Together, these factors lead to pulmonary edema by increasing the transcapillary pressure [15,16]. This disorder, which frequently manifests in hospital settings, is known to occur after extubation during the postoperative period. Anesthesiologists are typically the first to identify and treat this disorder, which occurs while patients are emerging from anesthesia in a state of light sedation. The disorder is categorized into two subtypes: Type I and Type II [[17], [18], [19]]. Type I NPPE, also referred to as laryngospasm-induced pulmonary edema, typically develops soon after acute airway obstruction, as in our patient. In comparison, type II NPPE develops after the resolution of chronic upper airway obstruction [20]. It is worth noting that the incidence of type I NPPE associated with postoperative acute upper airway obstruction is 9.6–12% compared to 44% of type II NPPE. Approximately 50% of adult NPPE events are due to postoperative laryngeal spasms [21]. Young, healthy, athletic patients seem at risk for this disorder because they can generate highly negative intrathoracic pressures during an obstructing event [22].

Characteristic chest radiographic findings consistent with the diagnosis include bilateral focal pulmonary infiltrates, extensive vascular pedicles, and a normal cardiothoracic ratio [4]. Another accurate and commonly used imaging modality is CT, which is gaining popularity for diagnosing this disorder, and its typical findings include accentuated consolidation with surrounding ground-glass opacity suggestive of pulmonary edema [23]. However, other abnormal and nonspecific patterns, such as “crazy paving,” which is common in pulmonary edema, may also be evident [24].

NPPE is generally a benign condition that can be quickly controlled if treated on time, and its symptoms may improve between 12 and 48 hours. As a general rule, the treatment of NPPE requires early recognition and is mainly supportive. The currently available treatment modalities emphasize the resolution of upper airway obstruction, which is the primary step, focusing on improving respiratory function and, reversing the pathophysiologic cascade, obviating pulmonary edema, and correcting hypoxemia [5,[24], [25], [26], [27], [28]]. The role of medications such as steroids, bronchodilators, and diuretics is still controversial, and they have shown contradictory results [6,24,29]. In our case, conservative treatment with supplemental oxygen was administered as 100% oxygen through mechanical ventilation and then via the nonrebreather face mask (flow 12 L/min) and initiation of intravenous diuretic (furosemide). The patient's symptoms of pulmonary edema improved rapidly, and this abrupt improvement of the patient's disease represents a typical case of NPPE.

We conducted a literature review on laryngospasm following dental abscess but found no article discussing this subject. In addition, the occurrence of NPPE in this case made it more uncommon. While there have been limited cases of NPPE in otolaryngology settings or maxillofacial surgeries, they usually occur during patient extubation or manipulation of the oropharyngeal area. However, our patient experienced laryngospasm without manipulating the oropharyngeal regions [7,8].

It should be noted that there were a couple of limitations in this case. Firstly, no head and neck imaging was performed to examine the dental abscess and pharyngeal condition. Secondly, the levels of inflammatory factors (such as ESR and CRP) were not monitored on a daily basis, which would have been ideal. These limitations could be attributed to the rare condition of the patient and the initial suspicion of a cardiogenic source of edema.

## 4 Conclusion

It is imperative to prioritize hygiene in oral surgeries. Any dental abscess or oral infection should not be neglected, as it may lead to dire consequences such as laryngospasm in rare cases. Although type I NPPE in cases of laryngospasm is rare, its burden is remarkable if left untreated. The main steps in its management are having a high index of clinical suspicion and early supportive treatment by maintaining a patent upper airway and administering supplemental oxygen. Mechanical ventilation may occasionally be needed for a brief period in severe cases. The role of diuretics and steroids is still a debate and requires further study.

## 5 Ethics statement

Written informed consent was obtained from the patient to publish this case report and accompanying images. A copy of the written consent is available for review by the Editor-in-Chief of this journal on request.

## Data availability statement

The data used in the study is available from the corresponding author upon reasonable request.

## CRediT authorship contribution statement

**Ali Hossein Samadi Takaldani:** Supervision, Methodology, Data curation, Conceptualization. **Kaveh Latifi:** Writing – original draft, Investigation, Formal analysis. **Asma Salmani:** Investigation, Data curation. **Mohammad Negaresh:** Writing – review & editing, Writing – original draft, Visualization.

## Declaration of competing interest

The authors declare that they have no known competing financial interests or personal relationships that could have appeared to influence the work reported in this paper.
